# A Comparative Feasibility Study for Transcranial Extracorporeal Shock Wave Therapy

**DOI:** 10.3390/biomedicines10061457

**Published:** 2022-06-20

**Authors:** Cyrill Slezak, Jonas Flatscher, Paul Slezak

**Affiliations:** 1 Department of Physics, Utah Valley University, Orem, UT 84058, USA; cslezak@uvu.edu; 2 Ludwig Boltzmann Institute for Experimental and Clinical Traumatology, AUVA Research Center, 1200 Vienna, Austria; e1416248@student.tuwien.ac.at; 3 Austrian Cluster for Tissue Regeneration, 1200 Vienna, Austria

**Keywords:** shock wave, transcranial, simulation, k-wave

## Abstract

The potential beneficial regenerative and stimulatory extracorporeal shock wave therapy (ESWT) applications to the central nervous system have garnered interest in recent years. Treatment zones for these indications are acoustically shielded by bones, which heavily impact generated sound fields. We present the results of high-resolution tissue-realistic simulations, comparing the viability of different ESWT applicators in their use for transcranial applications. The performances of electrohydraulic, electromagnetic, and piezoelectric transducers for key reflector geometries are compared. Based on density information obtained from CT imaging of the head, we utilized the non-linear wave propagation toolset Matlab k-Wave to obtain spatial therapeutic sound field geometries and waveforms. In order to understand the reliability of results on the appropriate modeling of the skull, three different bone attenuation models were compared. We find that all currently clinically ESWT applicator technologies show significant retention of peak pressures and energies past the bone barrier. Electromagnetic transducers maintain a significantly higher energy flux density compared to other technologies while low focusing strength piezoelectric applicators have the weakest transmissions. Attenuation estimates provide insights into sound field degradation and energy losses, indicating that effective transcranial therapies can readily be attained with current applicators. Furthermore, the presented approach will allow for future targeted in silico development and the design of applicators and therapy plans to ultimately improve therapeutic outcomes.

## 1. Introduction

Extracorporeal shock wave therapy (ESWT) is widely used [1] in the treatment of classic indications, including musculoskeletal disorders, e.g., plantar fasciitis [2], tendinopathies [3], and non-union fractions [4,5]. While interest has spread into new areas of soft tissue applications, including the field of urology, i.e., treatment of erectile dysfunctions [6], treatment sites are often directly accessible without acoustical obstruction. In contrast, treatments for indications such as meniscus tears [7], knee osteoarthritis [8,9], spinal canal treatment [10], or brain stimulation [11] require treatments in close proximity of (or through) the bone. The significant attenuations of applied shockwaves at bone interfaces pose challenges in treating the obstructed tissue.

Traditional ultrasound-based methods have taken on these challenges; clinical transcranial applications of the brain are already available. Ranging from basic imaging to invasive surgical methods (e.g., ExAblate), there is clear evidence that the transmission of controlled pressure waves through the skull is possible. The first attempts of applying shockwaves to Alzheimer’s patients [11] are now underway, harboring the potential of expanding the regenerative benefits of ESWT to the brain. In leveraging potential biomechanical effects on the cell membranes [12] to improve neuroplasticity, the impacts of a targeted (dorsolateral prefrontal cortex) or full brain treatment have been explored [11].

In order to evaluate the efficacy of transcranial ESWT treatments, experimental ultrasound data are only useful as a general guide for extrapolation; explicit shockwave propagation through the bone barrier has to be evaluated. In situ reference measurements in cadavers are not available and would only provide pointwise information rather than the extent of the sound field. Fortunately, increasing computational power and the development of new numerical tools allows for ultrasound simulations to predict wave propagation through the skull [13,14,15,16,17].

A computational approach to evaluating the transcranial ESWT sound fields (in particular, their dependence on the respective generating technologies and reflector geometries) is the only feasible way to investigate the fundamental nature of the bone barrier. Even a direct experimental comparison would be hampered by the multitude of mismatched distinguishing physical parameters across the manufacturers. This paper systematically compares the feasibility of a transcranial ESWT application using electrohydraulic (EH), electromagnetic (EM), and piezoelectric (PE) transducers and provides a side-by-side comparison of advantages and challenges for each applicator.

## 2. Materials and Methods

First, we created a set of representative in silico applicators for each generating technology and reflector design, which were matched on defined requirements to allow a systematic comparison. These were subsequently simulated in their use on a human head based on detailed CT images. A detailed comparison between the ESWT applicators’ simulated anatomical applications in comparison to a reference water bath one allowed for a multi-factorial evaluation for the feasibility of an effective transcranial treatment.

### 2.1. Experimental Setup

**Figure 1:**
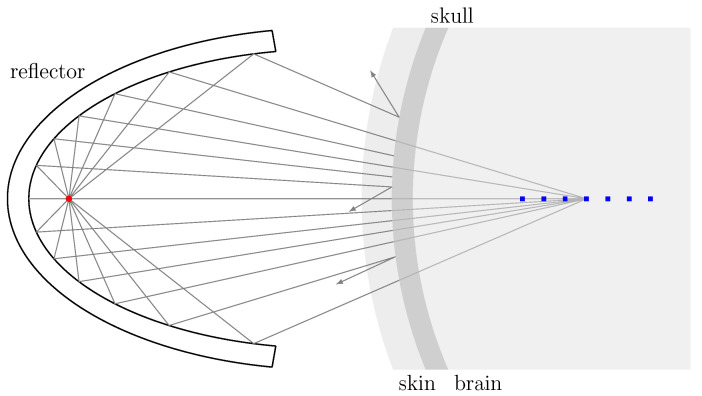
Representative cranial applicator placement, with the blue dots representing the multiple recording positions distributed within the focal zone.

A clinical transcranial ESWT application is modeled in silico. Figure 1 depicts the superficial cranial position of an applicator aligned with the longitudinal axis. The effectiveness of transmission within the phantom head was subsequently evaluated in comparing the resulting simulated sound field to that within a free-field water bath.

### 2.2. Transducers

Currently, three generating technologies (EH, EM, PE) for converting electrical energy into pressure waves are used for ESWT [18]. Based on the reflector design, intended use, energy range, and other parameters, it is difficult to compare technologies or manufacturers. A systematic comparison requires a set of fixed parameters. All our transducers were set to a nominal focal length of 45 mm and a maximum peak pressure of 20 MPa at the acoustic focal point. The latter, while 5 MPa less than the value suggested in Beisteiner et al. [11] for brain stimulation, was chosen (as it is a readily available peak pressure for many low/mid-energy devices). Regarding the safety of the use of shock waves in the brain, Beisteiner et al. [11] proposed a threshold of 0.25 ${\mathrm{mJ}/\mathrm{mm}}^{2}$ at 4 Hz with a maximal peak pressure of 25 MPa, but noted that below 40 MPa, no lesions had been observed. Additional safety concerns associated with the thermal index (TI) in ultrasound applications were negligible for shock waves due to the low pulse repetition rate.

With a nominal peak pressure set, corresponding applicator source waveforms have to be numerically determined for each applicator. Transducers in studies are often defined as 2D sources [19]; however, to model the characteristics of each transducer and study them in the presence of non-rotationally-symmetric obstacles (i.e., skull), it was necessary to remodel the sources in 3D. The source function for each applicator was adjusted, such that the resulting wave form at the focal point would match those of available reference applicators (detailed below). Subsequently, the reflector geometries were adjusted to the desired focal length and the source signal scaled to obtain a $p_{\max}=20\;\mathrm{MPa}$. The final sources were continuously placed at each voxel along a spherical shell (EH), cylindrical shell (EM), and spherical shell section (PE). For the EH applicator, the spherical source shell of radius r=0.5mm was centered at the primary focal point, mimicking the expanding plasma bubble, whereas a single voxel “spark” source would require excessively large pressure, which would make the simulation numerically unstable.

Electrohydraulic transducers were modeled for an ellipsoid reflector with a narrow focal spot and a parabolic reflector with a soft focal area. The two implemented EH transducers were based on the focused OE50 and the ‘soft and wide’ OP155 from MTS medical (MTS Medical; Konstanz, Germany). The electromagnetic (EM) transducer was based on the Storz DUOLITH SD1 with the ‘Sepia’ handpiece (Storz Medical AG; Tägerwilen, Switzerland). The respective waveforms were modeled on water bath reference measurements using the Müller-Platte Needle Probe PVDF hydrophone (Dr. Müller In-165 struments, Oberursel, Germany) and a digital storage oscilloscope (4-channel, 100 MHz bandwidth, DS1104Z Plus, Rigol, Beijing, China) for data collection.

In the implementation of piezoelectric transducers, two different designs were considered since the pressure levels of spherical transducers depend on their focusing angles [20,21]. The focusing strength is defined as

(1) $$F_{\#}=\frac{F}{2\;a_{0}}$$

where a low number corresponds to highly focused, and a high number to a weak focus with a wider focal zone. *F* is the radius of the curvature of the transducer and $a_{0}$ is the opening radius. Non-linearities were created either over a long distance at a lower pressure or at a shorter distance with higher pressure. This makes transducers with long focal zones at the same focus length, corresponding to a higher $F_{\#}$, favorable for generation in shock waves resulting in steeper gradients, but at the cost of energy loss [21]. To compare the resulting differences in their respective focal zone shapes, we considered two piezoelectric transducers with $F_{\#}=0.68$ based on the Swiss PiezoClast (Electro Medical Systems; Nyon, Switzerland)—the reference wave-form was based on the measurements provided by Sternecker et al. [22] and others [23,24,25,26] and a variant $F_{\#}=0.87$. Each transducer was implemented with piezoelectric elements in each surface voxel of the reflector. An overview of all referenced transducers can be found in Table 1.

#### Phase Correction

Phase correction is an ultrasound method for piezoelectric transducers where individual elements are phase-adjusted to yield the maximum barrier transmission. In this approach, PE ESWT devices should see improved signal form cohesion, energy, and peak pressure at the focal point. While phase correction is currently still technologically limited to the prototype high-intensity focused ultrasound (HIFU) [20] by (Imasonic, Voray Sur L’ognon, France) and ExAblate (HIFU) (InSightec; Tirat Carmel, Israel), this simulated approach for ESWT provides an upper-bound estimate for transmission.

This was implemented for the PE transducer with $F_{\#}=0.87$. The source signal and its input amplitude were left unaltered, but a phase correction was applied to each piezoelectric element. The corresponding individual peak-pressure time-delays were determined by replacing all the source voxels on the reflectors’ surfaces with sensors and recording the individual time delay of each voxel using an applied pressure at a single source voxel at the focal center.

### 2.3. Phantom

A computational 3D reconstruction of a human head was created by using available CT data from the Visible Human Project^®^. The CT data stem from a 72-year-old male patient with a planar pixel size of 0.489 mm and a slice thickness of 0.5 mm. The CT data were upscaled to the higher resolution of the simulation using the nearest-neighbor interpolation of the raw CT data.

**Figure 2:**
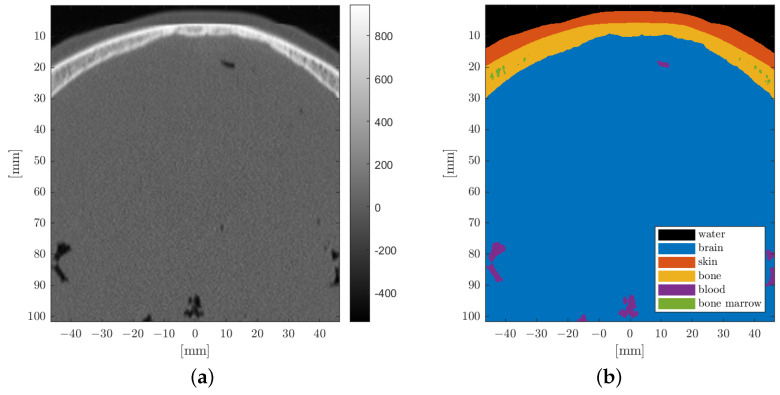
Cranial section of the CT image (**a**) and corresponding segmentation into the predominant tissue types (**b**). (**a**) CT, Hounsfield unit. (**b**) CT, clustered.

Density and the associated longitudinal speed of sound for each tissue type were assigned based on attenuation values utilizing the k-wave function hounsfield2density based on Schneider et al. [27]. The CT image was segmented into six different material types (water, skin, bone, brain, blood, bone marrow) as shown in Figure 2, based on a bone threshold HU value of 235 and the anatomical position.

#### 2.3.1. Attenuation

Reliable wave propagation simulations depend on the inclusion of representative acoustical parameters. Here, medium density ρ, speed of sound $c_{0}$, and attenuation α values were of primary importance. The pressure attenuation associated with a dampening of the signal was caused by density fluctuations being out of phase with the pressure wave fluctuations. For low wave frequencies, there were only modest energy losses and a slight dephasing, while at higher frequencies, the energy exchange could not keep up and the thermal losses increased [28].

The employed absorption coefficient α [dB/cm] is determined by the power law

(2) $$\alpha =\alpha_{0}\;\omega^{y}$$

where $\alpha_{0}$ [$\mathrm{dB}/({\mathrm{MHz}}^{y}\mathrm{cm})$] is the power law prefactor, ω the angular frequency, and *y* the power law exponent. For biological tissue, the power law exponent is found between 1 and 2 [29]; the absorption coefficient ranges between 0.1 and 60 dB/cm. The used attenuation values for soft tissues are found in Table 2.

In the literature, ‘absorption coefficient’ and ‘attenuation coefficient’ are frequently used synonymously. The absorption coefficient considers only the thermal losses through materials/particles, which are assumed to exhibit periodic fine microstructures, such as in the case for organic soft tissue [30]. The attenuation coefficient, on the other hand, includes all contributions to energy reduction at the measured resolution. In addition to the absorption at the microscopic level, influences due to the macrostructure of the material are taken into account, incorporating scattering effects. This is observed in low volume fraction bones (BV/TV—bone volume to total volume) where there is an increase in the scattering contribution to the total attenuation, while in high BV/TV bones, the absorption increases [31]. The thermal absorption coefficient is normally harder to determine and seldom found in the literature, while the attenuation coefficient is more common. However, for tissue with macroscopic homogeneity, the two parameters may be used interchangeably. The tissue (i.e., CT image) resolution used in this work was limited to a voxel size of 0.5 mm and, therefore, in a simulation, we favor soft tissue attenuation over absorption coefficients if both are available. For bone, on the other hand, this is not applicable at our resolution, and necessitates a more differentiated approach.

The non-linearity parameter *BonA* for most soft tissue is between 5 and 11 [32], while solids have a negligible ability to generate non-linearities. The singular power law exponent *y* in Equation (2) was chosen to be 1.2 as per reference [14]. This is due to the limitation in k-wave—that the power law exponent can only be specified for the whole volume and not independently for specific regions. An essential linear absorption for bone in the utilized spectral range was balanced against higher values for soft tissue, a higher value (see Table 2) and a compromise of y=1.2 was made. To increase the accuracy, the absorption power law prefactor $\alpha_{0}$ was recalculated by fitting it with the new power law exponent over the range of 0.4–3.0 MHz. If no power law exponent *y* was given, a value of 1 was assumed for this fitting process.

**Table 2:** Materials and their acoustic properties.

Material	$c_{0}$	ρ	$\alpha_{0}$	y	$\alpha_{0},\left(\mathrm{fitted}\right)$	B/A
	[m/s]	$\left[\mathrm{kg}/m^{3}\right]$	$\left[\frac{\mathrm{dB}}{{\mathrm{MHz}}^{y}\mathrm{cm}}\right]$		$\left[\frac{\mathrm{dB}}{{\mathrm{MHz}}^{1.2}\mathrm{cm}}\right]$	
Soft tissue [29]	1500	1000	0.43	-	0.37	-
Skin [33]	1590	1065	1.79 [34]	0.87 [34]	1.38	7.9 [35]
Connective tissue [35]	1613	1120	1.57	-	1.34	-
Muscle [35]	1547	1050	1.09	-	0.93	7.5 [36]
Fat [33]	1450	950	0.6	1	0.51	10
Skull bone [29]	2900	1800	14.77	0.93	11.91	-
Brain [33,34]	1550	1030	0.8	1.35	0.9	6.9
Blood [33,35]	1584	1060	0.15	1.21	0.15	6.1
Bone marrow	1680	1150	8	1.2	8	6
Water [33,37]	1482	998	2.17 × 10${}^{-3}$	2	4.24 × 10${}^{-3}$	5.2
Degassed water [38]	-	-	-	-	-	4.8
Air	343	1.2	1.62	-	-	-
Steel	5750	8030	-	-	-	-
PVC	2400	1380	-	-	-	-

#### 2.3.2. Bone Attenuation Models

Due to the importance and dominance of the transmission losses associated with the skull, we employed a refined bone model. While soft tissue mostly supports the propagation of compression waves and little to no shear waves, bone supports both. Additionally, the aforementioned bone structure and, therefore, its relevant physical parameters for use in the simulation, are much more heterogeneous. While much of the attenuation is caused by microstructures inside the bone [39], these were beyond the scale captured in our CT data.

The ranges for density and speed of sound within bone is considerably wider than for the present soft tissue types, which were respectively assumed to be homogeneous and, thus, described by a single attenuation value. The differences between using the heterogeneous values versus assuming a homogeneous skull can be quite high and differences of up to 50% in pressure were observed [17]. In choosing a singular representative value, those of cortical bone are preferable over cancellous bone or skull density-averaged ones [40]. To test the influence of the bone, three different attenuation models were implemented.

#### 2.3.3. Homogeneous Attenuation

This represents the simplest approach, where the density and speed of sound were generated by hounsfield2density; the absorption power law prefactor value $\alpha_{0}=11.91$$\frac{\mathrm{dB}}{{\mathrm{MHz}}^{1.2}\mathrm{cm}}$ according to [29] was assumed homogeneous over the whole density interval.

In contrast, the approaches by McDannold et al. [39] and Pichardo et al. [41] attempted to map different attenuation and speed of sound values to each density value on the interval, according to an interpolation of the experimental data.

#### 2.3.4. Heterogeneous Attenuation acc. to McDannold

In this mode, the density conversion curve was adjusted. A linear relationship between the Hounsfield units (HU) and density was assumed, with HU = −1000 and HU = 57 representing air and soft tissue. Assuming a density of 1.2 ${\mathrm{kg}/m}^{3}$ and 1030 ${\mathrm{kg}/m}^{3}$, this resulted in a linear equation of

(3) ρ=0.97HU+975.2.

**Figure 3:**
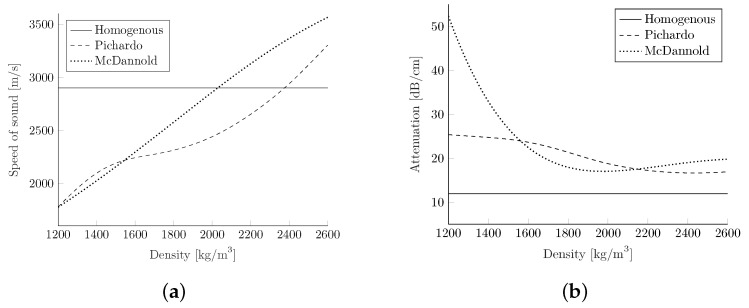
Comparison of the speed of sound (**a**) and attenuation power law prefactor $\alpha_{0}$ (**b**) at 1 MHz based on [39,41]. (**a**) Speed of sound. (**b**) Attenuation.

While applying the model directly yields the speed of sound, the attenuation mapping had to be adapted. As the original was based on results using a 660 kHz transducer, it was converted into a k-wave-required 1 MHz curve by using the power law component of 0.93, specified by Mohammadi et al. [29]. Afterwards the curve was fit to a power law exponent of 1.2 on the range of 0.4–3 MHz, as well as all other attenuation parameters. The conversion curves are shown in Figure 3.

#### 2.3.5. Heterogeneous Attenuation acc. to Pichardo

In Pichardo et al. [41], the density values are calculated with a linear correlation of

(4) ρ=HU+1000

with the assumptions of an air density of 0 ${\mathrm{kg}/m}^{3}$ (HU = −1000) and a water density of 1000 ${\mathrm{kg}/m}^{3}$ (HU = 0). The attenuation coefficient and speed of sound mapping were carried out for several different frequencies, but only the attenuation values at 1 MHz can be used by k-wave as input. To generate the conversion curve for the speed of sound as before, a 2D interpolation was applied. The attenuation values were interpolated by using a spline interpolation in the dimension of the density, and the $\alpha_{0}\;f^{1.2}$, in the frequency dimension. The resulting values are depicted in Figure 3.

### 2.4. Simulation

The Matlab open-source toolbox k-wave, a k-space-based pseudo-spectral method, was shown to create equal or better accuracy than comparable finite-difference time domain methods [16] for ultrasound simulation. While finite-difference time domain methods require at least six to ten grid points per element and have to be used for a reliable result, according to the Nyquist theorem, only two grid points per wavelength (PPW = λ/Δx) are required with k-wave, allowing for a coarser grid resolution Δx. This reduces the number of grid points in a 3D simulation by at least a factor of $3^{3}$, allowing for modestly-sized three-dimensional grid sizes to become numerically feasible.

Ultimately, a high resolution is necessary to depict the shock wave; a maximal computationally limited domain size of 1024×648×648 was chosen with a maximal voxel size of 0.17 mm. Due to the periodic nature of the k-space method, an additional 10-voxel deep perfectly-matched layer (PML) provides sufficient absorption without reflection at the grid boundaries. This resolution allows for frequencies up to 4 MHz and reduces to a frequency below 1.3 MHz with the six required points per wavelength at a medium interface. The time steps were determined by using a CFL number of 0.3; the k-wave required reference speed of sound was set to the tissue speed of sound for the highest accuracy in the observed domain.

Computations were completed on an Intel Server at the Center for High Performance Computing (CHPC) in Utah with 64 GB of RAM; the average computation time on an Intel Xeon E5-2670 v2 (10 CPU cores) was about 30 h.

### 2.5. Signal Recording

Wave forms of the pressure signal were recorded by averaging the pressure values over a circle with a radius of 0.25 mm mimicking a needle-type hydrophone. This choice was made as in situ measurements would rely on the rigidity of the hydrophone, which cannot be provided by more accurate laser hydrophones. Peak compression and tensile pressures were recovered from the entire spatial domain while pressure-time curves were restricted to individual locations of interest (i.e, geometric and acoustical focuses). The shock front pressure gradient of the waveforms $\nabla p=\frac{\Delta p}{\Delta t}$ was determined between 20% and 80% of the maximal peak pressure of the signal. FFT spectral analyses of the pressure signals were calculated from ‘zero padded’ inputs, which were zero-filled until 10 times the observed signal length was reached.

## 3. Results

**Figure 4:**
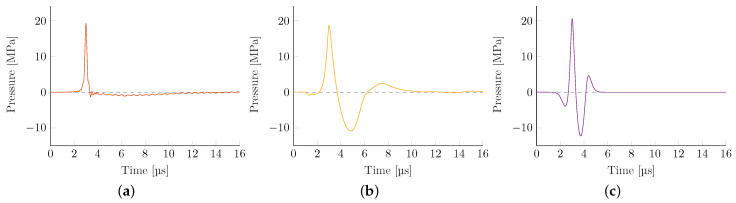
Representative simulated pressure-time curves in the water bath at the respective acoustic focus. Changes in reflector geometry for the same generating technology yield no visually discernible differences. (**a**) Electrohydraulic (elliptical). (**b**) Electromagnetic. (**c**) Piezoelectric ($F_{\#}=0.68$).

The source pressure variations of each applicator technology and respective geometry were adjusted to yield comparable peak pressures of 20 MPa in initial open-field reference simulations in a water bath. Figure 4 depicts representative pressure-time curves evaluated at locations along the transducer axis where the highest peak-pressure $p_{+,max}$ was observed (acoustical focus). These far-shifted locations of peak pressures (away from the geometric focus) are due to the focusing reflection at not-normal angles of incidence being elongated, dependent on both the reflector geometry and local pressure levels. Table 3 summarizes the resulting envelope parameters, peak compression $p_{+,max}$, and tensile $p_{-,max}$ pressures, the shock front gradienpressures, the shock front gradient ∇p, and the energy flux density PII.

There are some observable key differences between the applicator technologies. The local energy flux density of EH transducers is significantly smaller than other technologies. Since all peak pressures have been made comparable, the resulting wave has a substantially lower tensile wave and pulse width (full-width half-maximum). The latter in particular is closely tied to the shock front pressure gradient, observed to be the largest in the EH devices, which is the result of re-focusing an initial shock wave that maintains a steep shock front gradient throughout. However, for the case of the weakly-focused parabolic EH, the excessively large source pressure required to obtain the targeted 20 MPa focal pressure far exceeds that of any other simulation, and the associated gradient results in the onset of numeric instabilities at the achievable grid size. This likely yields an underestimation of the gradient and derived quantities.

The EM transducer shows the lowest gradient while having the highest PII due to its long duration and low generating frequency. Both PE applicators show similar properties with only minor variations. The transducer with the $F_{\#}=0.87$ shows the expected higher gradient at the associated loss of tensile pressure and PII.

**Figure 5:**
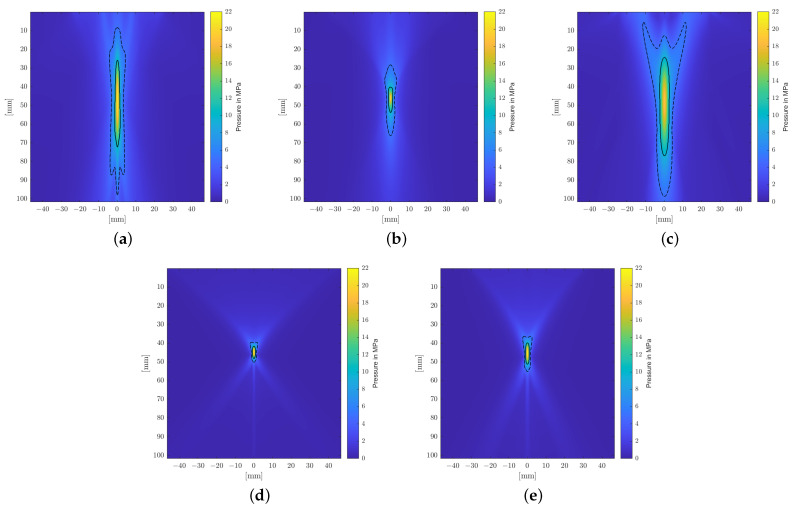
Longitudinal cross-section of the sound field showing peak pressure in a reference water bath. The −6 dB and 5 MPa focal zones are delineated by solid and dashed lines. (**a**) EH, parabolic. (**b**) EH, elliptical. (**c**) EM. (**d**) PE, $F_{\#}=0.68$. (**e**) PE, $F_{\#}=0.87$.

A longitudinal cross-sectional visualization of the positive peak pressures of the sound fields is shown for each applicator in Figure 5. It clearly shows the narrow, point-like focal zones of the PE transducers due to the self-focusing spherical arrangements of the piezoelectric elements. Comparably compact focal zones can be achieved using refocusing EH elliptical reflectors, but weakly focusing parabolic EH and EM reflectors allow for extended treatment zones.

**Figure 6:**
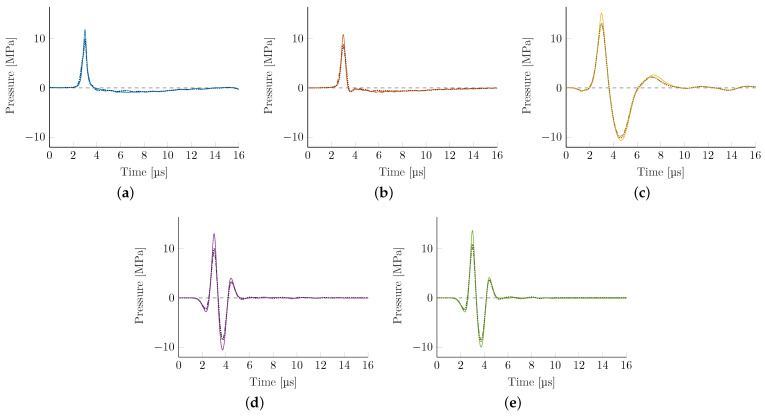
Waveforms at the acoustical focus for the different bone attenuation models (solid line = homogenous, dashed line = McDannold [39], dotted line = Pichardo [41]). The latter two are mostly overlapping and almost indistinguishable. (**a**) EH, parabolic. (**b**) EH, elliptical. (**c**) EM. (**d**) PE, $F_{\#}=0.68$. (**e**) PE, $F_{\#}=0.87$.

Introducing a human head phantom into the simulation allowed us to systematically evaluate the impact of the skull bone and the surrounding tissue on the propagation, focusing on the sound field. Throughout, attenuation values for soft tissue were held constant (as they play a minor role); the different attenuation models for the acoustically dominant bone are compared. Figure 6 shows the attenuated pressure-time graphs at the acoustical focus for each of the three bone models. There is a marked loss in peak pressure and associated energy flux density but the general waveforms are mostly preserved.

Taking a closer look at the envelope parameters of these wave-forms in Table 4, we find some consistent trends. There is a marked reduction of maximal peak pressure, which, depending on the transducer, may only reach 50% of the reference water bath values while tensile pressure components are generally less affected. The associated pressure gradient of the shock front is commensurately also significantly reduced for all technologies with EH transducers being most affected. This is associated with the non-linear signal attenuation at the skull, which acts as a low pass filter, resulting in a comparable pressure gradient across all applicators. The energy flux density is reduced, with the EM transducer being able to maintain the most energy flux density while propagating through the skull.

**Figure 7:**
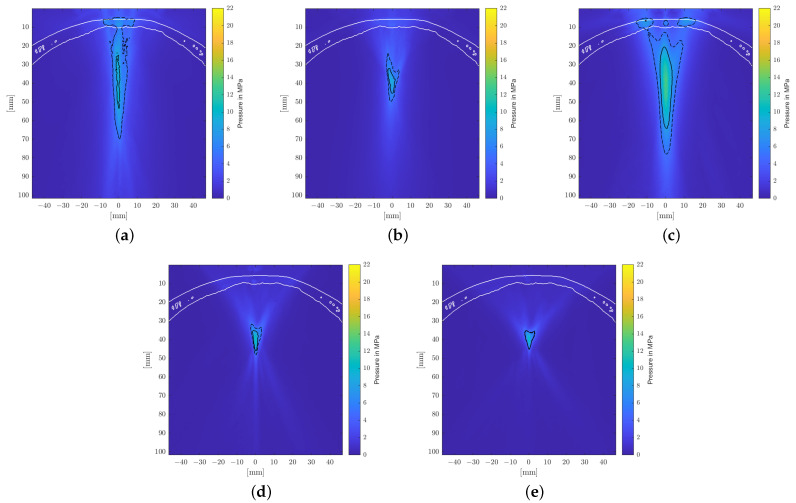
Longitudinal section of the maximal pressure in the transcranial application, using the Pichardo [41] attenuation model. The −6 dB and 5 MPa focal zones are delineated by solid and dashed lines. (**a**) EH, parabolic. (**b**) EH, elliptical. (**c**) EM. (**d**) PE, $F_{\#}=0.68$. (**e**) PE, $F_{\#}=0.87$.

Figure 7 shows the pressure distribution inside the skull based on the model suggested by Pichardo et al. as a suitable compromise between the other two models. Clearly discernible differences in the water bath results can be categorized into effects associated directly with the employed attenuation model and those attributable to the bone shape and inhomogeneities.

The observed displacement of the focal zone is similar in all cases and is mainly associated with the skull’s geometry and not the attenuation coefficient. The more dense skull acts as an acoustic lens [17,42] and moves the focus closer to the skull. In the chosen geometry, this near-shift essentially nullifies the previous far-shift associated with increased angles of reflection at the reflectors due to the high pressures. The result is that the transcranial focal point location comes to lie very close to the geometric focus for each applicator. Note however that this is due to the particular relative placement of the reflector and the skull’s curvature. Changes in the position and/or incident angle of the transducers on the other hand will move the focal point location again.

Figure 7 shows the maximum peak pressure map fixed to the same relative position to the applicator as within the water bath. In addition to the focal shift, there is a significant impact on the shape of the focal zone. For the transducers with smaller focal zones, in particular, we noticed clearly visible non-symmetric distortion. The position of the skull is clearly discernible due to the pressure build-up in the bone.

**Figure 8:**
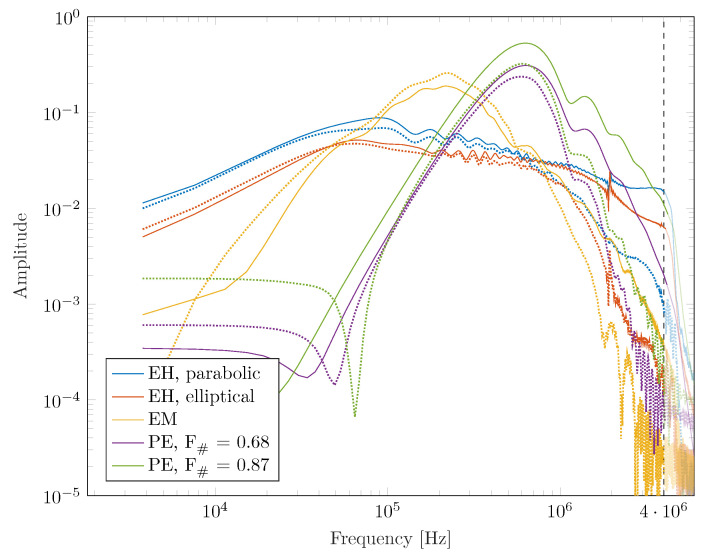
Fourier transformed waveforms at the acoustic focus in the empty water bath (solid) compared to the transcranial model, utilizing the attenuation model by Pichardo [41] (dotted).

In addition to the spatial modulation of the sound field, there is an observable impact on the frequency weighting with each pulse, which provides additional insight. Figure 8 shows the Fourier spectrum of the water bath waves in contrast to the Pichardo et al. attenuated ones inside the skull. A general fall-off towards higher frequencies is observed, associated with the high-frequency filtering of the bone and reduced focusing due to inhomogeneities while low frequencies are well transmitted.

### Phase Correction

**Figure 9:**
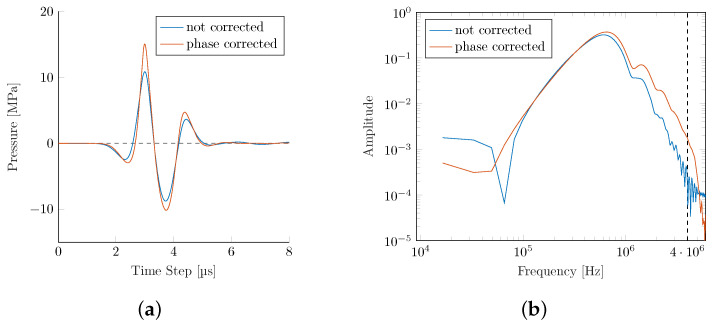
Comparison of the pressure signals and their frequency spectra for the non-corrected and phase-corrected PE transducers. (**a**) Signal pressure. (**b**) Frequency spectrum.

Two of the key challenges identified in intracranial applications are (1) the predominant absorption of higher frequencies within the bone, and (2) reflection and dephasing at the bone interface. In a technologically limited (due to the element size and individual time-delay limitations in commercial applicators), but interesting best-case scenario, we explore the achievable upper bounds for the wave transmission within a phase-corrected PE. Simulating a phase-corrected PE $F_{\#}=0.87$ transducer utilizing the Pichardo attenuation model leads to a significant recovery of pressure losses (see Figure 9 and Table 5).

The phase correction results in an improved peak pressure as fewer high-frequency components are lost during bone transmission. This results in a significantly steeper gradient and higher PIIs. The focal center is shifted even closer to the actual geometric center than the PE simulation in the water bath (see Table 5).

**Figure 10:**
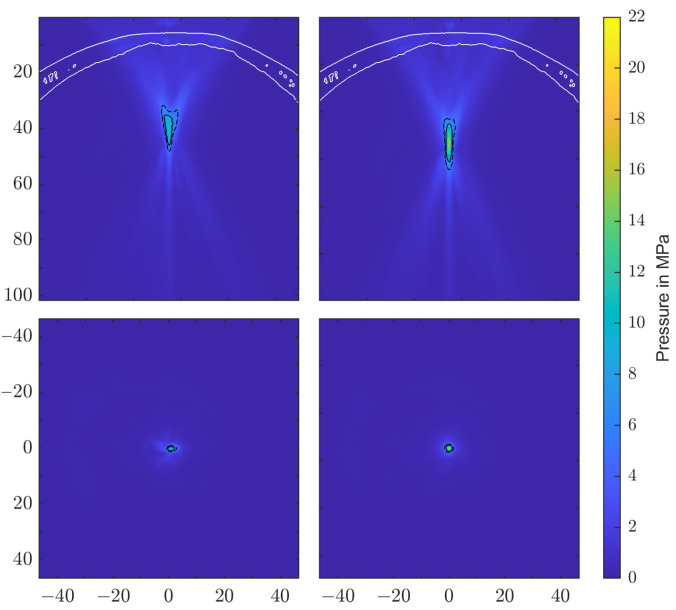
Comparison of longitudinal (**top**) and cross-sectional (**bottom**) transcranial pressure zones for the non-corrected (**left**) and phase-corrected simulations (**right**).

At the same time, we see improvements in the maximum peak pressure plot in Figure 10. While the phase-corrected simulation retains some asymmetry over the reference simulation in the water bath, it is a clear improvement over the non-corrected PE transducer.

## 4. Discussion

This study provided the first direct comparison of the viability of different ESWT technologies to be used for transcranial applications based on high-resolution numerical simulations. Our fixed focal length and peak pressure normalized applicators yielded similar free-field (i.e., water bath) sound fields as their comparable commercial devices. The evaluation of the pressure waves inside the skull shows that all technologies can successfully treat at clinically relevant energies and pressures, even though application parameters may have to be increased to maintain soft tissue levels. Peak pressures are reduced by less than 40%, except for PE devices with smaller focusing strengths and, consequently, slightly more attenuated energy flux densities. Waveforms retained their overall shape, but noticeable drops in shock front gradients were observed. These results are in line with initial point-wise experimental measurements for select applicators on cadavers [11], and will be, without a doubt, extended to others. To gain a better understanding of the qualitative differences in the transcranial field attenuation due to the respective technologies involved, we have to consider the limitations of the numerical simulation approach.

One limiting parameter is the spatial resolution and discretization of the bone interface. At crucial medium discontinuities, especially at non-planar interfaces due to stair-casing effects [16], more PPW are required to obtain an error of ≤10% in the intensity amplitude. In a convergence model in 3D with all numerical errors included, 6 PPW seem to be sufficient in homogeneous media. Mostly, the pressure amplitude (and not the location) is affected; the latter does not vary more than 50% of the wavelength and is suggested to be solely due to the misregistration of source points [16]. Therefore, for spatial targeting, a lower resolution approach may indeed be sufficient.

We investigated three different bone attenuation models in detail, but while being reliable for soft tissue applications, neglecting the shear modulus for the bone may not be appropriate. While shear wave solvers exist, the computational demands for a full grid simulation at sufficiently high resolutions are prohibitive. Mueller et al. [17] reported a deviation of 15% in the pressure amplitude, focal volume size, and location, including shear waves. However, as shown by Treeby et al. [43], the shear waves are negligible below a critical angle of incidence around $34^{\circ }$ to the normal of a surface. In another study [44], it was determined that the critical angle depends on the density and structure of the bone, and a critical angle between $30^{\circ }$ to $45^{\circ }$ was observed. Due to the small aperture sizes of the applicators and the low cranial curvature, the angles of incidence of the relevant pressure waves are well below the critical angles, as can be seen in Figure 7.

There is an indication in our data that a correct bone attenuation model is essential. We see consistent results in the models by McDannold et al. and Pichardo et al., while an assumption of a homogeneous attenuation for the entire bone yields an expected overestimation of the pressure strengths. An additional important factor impacting the transmitted pressure waves is the skull bone composition. Varying individual anatomic layer thicknesses and microstructures affecting mechanical properties [45] may further attenuate the sound fields in full head simulations but are not expected to impact applicator comparisons. While there are small differences in the pressure waves and associated sound fields for the two differentiating models, we applied Pichardo’s attenuation model for further analysis.

We saw a consistent focusing effect of the skull bone manifesting itself in a near-shift of the acoustic focal point, which was comparatively small to the overall focal length of the reflector. This is noteworthy since the elongated focal volumes, as determined by the geometry of the reflector and not the bone interface, remained intact, allowing for deep treatment zones. In the generated pressure fields there was a marked increase in attenuation for both EH applicators over the other technologies. In contrast to other methods, EH shock wave generation is based on the refocusing of source waves, which are inherently already shock waves. To adequately capture their steep pressure gradients, a sufficiently high-frequency threshold is required. While our grid resolution supports up to a 4 MHz signal in soft tissue, this is drastically reduced within the bone due to the higher density and corresponding wave speed. As a result, those high-frequency contributions and, consequently, associated energies, are not accounted for in the simulation (see Figure 8) and can only be partially regained by a non-linear buildup of the shock front in the vicinity of the focal point. We would expect this to be comparable to EM and PE devices, which are still dominated by longer wavelengths as they penetrate the skull. This can be potentially mitigated by employing a grid scaffolding [46] about the bone and thereby locally increasing the spatial resolution. Due to a significant increase in computational resources, this is outside the scope of this paper and will be the subject of future EH investigations.

In conclusion, we saw high ESWT transmission rates for transcranial applications for all technologies. Long-wavelength source-based technologies (EM) yield the highest peak pressures and energies and the most reliable computational results due to low bone attenuation. De-focusing further reduces peak pressures, especially for highly focused reflectors, but can potentially be remedied via PE phase corrections. In contrast, the presented EH results are expected to constitute lower bound estimates due to computational limitations resulting in limiting high-frequency components within the bone. In general, transducers with bigger focal zones and lower frequency spectral weights have an advantage in maintaining maximal pressure and energy. Therapeutically relevant and sound field geometries, spatial extents, and attenuated volumes are generally preserved and can be readily adjusted by the choice of generating technology and the reflector designed and planned in silico, as presented in this work.

## Figures and Tables

**Table 1:** Comparison of the model transducers’ focal zones fz, with their apertures and focal lengths as references.

Material	fz Length	fz Width	Focal Length	Aperture
	[mm]	[mm]	[mm]	[mm]
MTS OE50 (EH)	23.3	5.7	∼30	∼45
MTS OP155 (EH)	73.45	7.4	∼45	∼50
DUOLITH SD1 ‘Sepia’ (EM)	32	3.9	50	∼45
PiezoClast (PE)	9.55	2	45	100

**Table 3:** Simulated positive and negative peak pressures, shock front gradient, and energy flux density in a water bath for the different applicators. Measurements taken at the acoustical focus.

Transducer	$p_{+,\max}$	$p_{-,\max}$	$\frac{\Delta p}{\Delta t}$	PII
	[MPa]	[MPa]	[Pa/s]	[$\mathrm{mJ}/{\mathrm{mm}}^{2}$]
EH, parabolic	21.25	−1.94	1.62 × 10${}^{14}$	0.0438
EH, elliptical	20.09	−1.48	1.11 × 10${}^{14}$	0.0418
EM	19.04	−11.92	3.61 × 10${}^{13}$	0.1990
PE, $F_{\#}=0.68$	21.82	−12.59	8.01 × 10${}^{13}$	0.1236
PE, $F_{\#}=0.87$	21.47	−10.79	9.86 × 10${}^{13}$	0.1020

**Table 4:** Transcranial maximum and minimum pressure, gradient, and energy flux density of the simulation as a percentage of the water bath reference value for each attenuation model and applicator. The last column provides the translation in the direction of the axis $t_{A}$ and the x-y plane $t_{x}$, $t_{y}$ from the acoustic focus in the water bath.

*(a)* Homogenous attenuation coefficient					
Applicator	$p_{+,max}$	$p_{-,max}$	$\frac{\Delta p}{\Delta t}$	PII	$d_{A}$/ $d_{x}$/ $d_{y}$
	[%]	[%]	[%]	[%]	[mm]
EH, parabolic	85.18	124.23	18.70	64.84	−1.36/−0.17/1.02
EH, elliptical	71.28	87.16	26.04	61.00	−2.72/−0.34/0.68
EM	87.34	100.67	63.16	89.65	−1.09/0.00/1.02
PE, $F_{\#}=0.68$	61.23	85.86	49.81	59.47	−3.57/0.00/0.00
PE, $F_{\#}=0.87$	71.50	97.31	46.04	70.29	−3.91/−0.34/0.51
*(b)* Attenuation model following McDannold et al. [39]					
Applicator	$p_{+,max}$	$p_{-,max}$	$\frac{\Delta p}{\Delta t}$	PII	$d_{A}$/ $d_{x}$/ $d_{y}$
	[%]	[%]	[%]	[%]	[mm]
EH, parabolic	72.61	213.40	9.81	50.68	−1.36/−0.17/1.36
EH, elliptical	60.63	90.54	15.23	44.02	−2.72/−0.34/0.85
EM	78.31	90.27	47.92	75.08	−1.09/0.00/1.19
PE, $F_{\#}=0.68$	44.73	70.14	30.71	34.55	−4.42/−0.17/0.34
PE, $F_{\#}=0.87$	60.41	88.51	28.80	45.98	−4.08/−0.17/0.68
*(c)* Attenuation model following Pichardo et al. [41]					
Applicator	$p_{+,max}$	$p_{-,max}$	$\frac{\Delta p}{\Delta t}$	PII	$d_{A}$/ $d_{x}$/ $d_{y}$
	[%]	[%]	[%]	[%]	[mm]
EH, parabolic	80.33	191.24	10.99	53.42	−1.36/−0.17/1.19
EH, elliptical	65.60	103.38	21.26	47.85	−2.72/−0.34/0.85
EM	80.36	93.37	50.69	78.99	−1.09/0.00/1.19
PE, $F_{\#}=0.68$	48.12	72.76	36.20	38.27	−5.44/0.17/0.17
PE, $F_{\#}=0.87$	64.79	93.23	32.76	51.76	−4.93/0.00/0.68

**Table 5:** Comparison of the PE $F_{\#}=0.87$ transducer’s acoustical focal pressures with and without the phase correction method; the empty water bath simulation serves as a reference.

Transducer	$p_{+,\max}$	$p_{-,\max}$	$\frac{\Delta p}{\Delta t}$	PII
	[MPa]	[MPa]	[Pa/s]	[$\mathrm{mJ}/{\mathrm{mm}}^{2}$]
Water bath	21.47	−10.79	9.86 × 10${}^{13}$	0.1020
Phase correction	15.54	−10.29	5.98 × 10${}^{13}$	0.0762
Non-corrected	13.91	−10.06	3.23 × 10${}^{13}$	0.0528

## Data Availability

The data and underlying code presented in this study are available on request from the corresponding author. The data and code are not publicly available due to potential conflicts of confidentiality.
